# Lung tumor exosomes induce a pro-inflammatory phenotype in mesenchymal stem cells via NFκB-TLR signaling pathway

**DOI:** 10.1186/s13045-016-0269-y

**Published:** 2016-04-18

**Authors:** Xiaoxia Li, Shihua Wang, Rongjia Zhu, Hongling Li, Qin Han, Robert Chunhua Zhao

**Affiliations:** Center of Excellence in Tissue Engineering, Key Laboratory of Beijing, Institute of Basic Medical Sciences and School of Basic Medicine, Chinese Academy of Medical Sciences and Peking Union Medical College, Beijing, People’s Republic of China; Center of Translational medicine Peking Union Medical College Hospital, Chinese Academy of Medical Sciences and Peking Union Medical College, Beijing, People’s Republic of China

**Keywords:** Exosomes, MSCs, Tumor-supportive, Inflammation, NF-κB, TLR2, HSP70

## Abstract

**Background:**

In tumor microenvironment, a continuous cross-talk between cancer cells and other cellular components is required to sustain tumor progression. Accumulating evidence suggests that exosomes, a novel way of cell communication, play an important role in such cross-talk. Exosomes could facilitate the direct intercellular transfer of proteins, lipids, and miRNA/mRNA/DNAs between cells. Since mesenchymal stem cells (MSCs) can be attracted to tumor sites and become an important component of the tumor microenvironment, there is an urgent need to reveal the effect of tumor exosomes on MSCs and to further explore the underlying molecular mechanisms.

**Methods:**

Exosomes were harvested from lung cancer cell line A549 and added to MSCs. Secretion of inflammation-associated cytokines in exosome-treated MSCs were analyzed by RT-PCR and ELISA. The growth-promoting effect of exosome-treated MSCs on lung tumor cells was evaluated by in vivo mouse xenograft model. Signaling pathway involved in exosomes-treated MSCs was detected by PCR array of human toll-like receptor signaling pathway, RT-PCR, and Western blot.

**Results:**

Data showed that lung tumor cell A549-derived exosomes could induce a pro-inflammatory phenotype in MSCs named P-MSCs, which have significantly elevated secretion of IL-6, IL-8, and MCP-1. P-MSCs possess a greatly enhanced ability in promoting lung tumor growth in mouse xenograft model. Analysis of the signaling pathways in P-MSCs revealed a fast triggering of NF-κB. Genetic ablation of Toll-like receptor 2 (TLR2) by siRNA and TLR2-neutralizing antibody could block NF-κB activation by exosomes. We further found that Hsp70 present on the surface of lung tumor exosomes contributed to the induction of P-MSCs by A549 exosomes.

**Conclusions:**

Our studies suggest a novel mechanism by which lung tumor cell-derived exosomes induce pro-inflammatory activity of MSCs which in turn get tumor supportive characteristics.

**Electronic supplementary material:**

The online version of this article (doi:10.1186/s13045-016-0269-y) contains supplementary material, which is available to authorized users.

## Background

Tumor microenvironment has a close relationship with tumor development and metastasis [1]. A complex milieu of non-malignant cells compose tumor microenvironment, contributing to tumor progression by interactions with tumor cells and/or with each other [2]. Mesenchymal stem cells (MSCs) are an important component of these cells. MSCs are defined as multipotent stem cells that have the capacity to give rise to adipocytes, osteoblasts, and chondrocytes [3]. They can be isolated from a number of tissues including bone marrow, adipose tissue, and umbilical cord blood. Although the function of naïve MSC in tumor remains controversial, the tumor-supporting roles of tumor associated mesenchymal stem cells have been acknowledged [4]. This may be attributed to a long-time “education” by tumor cells.

Tumor cells can modulate tumor stromal cells through intercellular communications. This process can be mediated through direct cell contact or through secreted signaling factors (cytokines, chemokines, and growth factors) and microvesicles. Exosomes, one kind of membrane vesicles containing proteins, mRNA, miRNAs, and DNAs, can be produced by tumor cells as well as other various cell types [5]. Recently, exosomes in tumor microenvironment are attracting more and more attention. They are shown to be small particles but big players in cancer progression and metastasis [6–8] as cancer cells could reprogram surrounding stromal cells into tumor supportive myofibroblasts through secreted exosomes. Data showed that exosomes from ovarian and breast cancer cells can convert adipose-derived MSCs (AMSC) into myofibroblast-like cells [9, 10]. Bone-marrow MSC (BMSC) could also be triggered to differentiate into pro-angiogenic and pro-invasive myofibroblasts by prostate cancer cell-derived exosomes [11]. Exosomes released by chronic lymphocytic leukemia cells could induce the transition of stromal cells into cancer-associated fibroblasts [12].

While most studies concentrate on reprogramming MSCs into tumor-supportive myofibroblasts by various cancer cell-derived exosomes, few studies have paid attention to the immuno-phenotype changes of MSCs after incubation with tumor cells derived exosomes. Tumors are regarded as chronic injuries that are difficult to heal [13], and inflammation plays an important role in tumorigenesis, tumor progression, and metastasis [14]. Besides, MSCs are known for its immunomodulatory capacity through secreting related factors such as cytokines [15]. Thus, there is an urgent need to dictate the immuno-phenotype changes of MSCs in tumor microenvironment.

Previous research about MSCs immuno-phenotype changes often use pre-conditioned MSCs with inflammatory factors. Results show that long-term stimulation with TNF-α, IFN-γ, and other factors could upregulate the expression of various pro-inflammatory genes in MSCs [16]. The team of Yufang Shi noticed TNFα-pretreated BM-MSCs mimicked lymphomas-MSCs in their chemokine production profile and ability to promote tumorigenesis of lymphoma, melanoma, and breast carcinoma [17]. Therefore MSCs that have elevated secretion of inflammatory factors show great relevance with tumor progression.

In the present study, we will investigate whether exosomes from lung tumor cells could affect immuno-phenotype of AMSCs and try to reveal the molecular mechanisms involved in this process. Since few studies have elucidated the mechanisms by which lung cancers influence AMSC through exosomes and lung cancer is one of the leading causes of cancer-related death which incline to transfer to other sites such as bone through tumor-stromal interactions in late stage [18], it is meaningful to explore this question.

## Results

### Lung tumor cell A549-derived exosomes are actively incorporated by MSCs

**Fig. 1:**
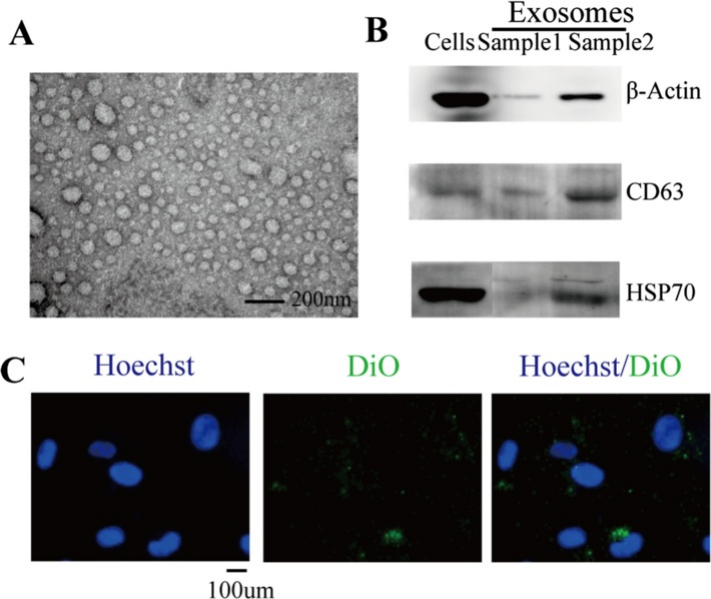
Characterization of exosomes secreted by A549 cancer cells. **a** Electron micrograph of exosomes derived from A549 cancer cells. *Scale bar* = 200 nm. **b** Detection of HSP70and CD63 expression in exosomes by Western blot. A549 cells lysate was used as positive control with loading mass of 20 μg total proteins. Samples represent A549 exosomes. Sample 1 was 1 μg loaded. Sample 2 was 10 μg. **c** Internalization of DiO-labled A549 cancer cells derived exosomes at 2 h in MSCs. *Scale bar* = 100 um

Exosomes were isolated from serum-free culture medium of A549 cells through a series of centrifugation and filtration steps. Under transmission electron microscope, these exosomes were observed to be cup-shaped vesicles of approximately 30–120 nm in diameter (Fig. 1a). In addition, CD63 and HSP70, the protein markers of exosomes, were also detectable (Fig. 1b). Since exosomes could be labeled by lipophilic cell tracking dyes, DiO-labeled exosomes were added to MSCs serum-free culture medium. Exosomes uptake was observed 2 h after application (Fig. 1c) and accumulated over time (data not shown). These data demonstrate that MSCs can efficiently internalize A549 cell-derived exosomes.

### A549 cell-derived exosomes significantly stimulate inflammatory cytokine production in MSCs

**Fig. 2:**
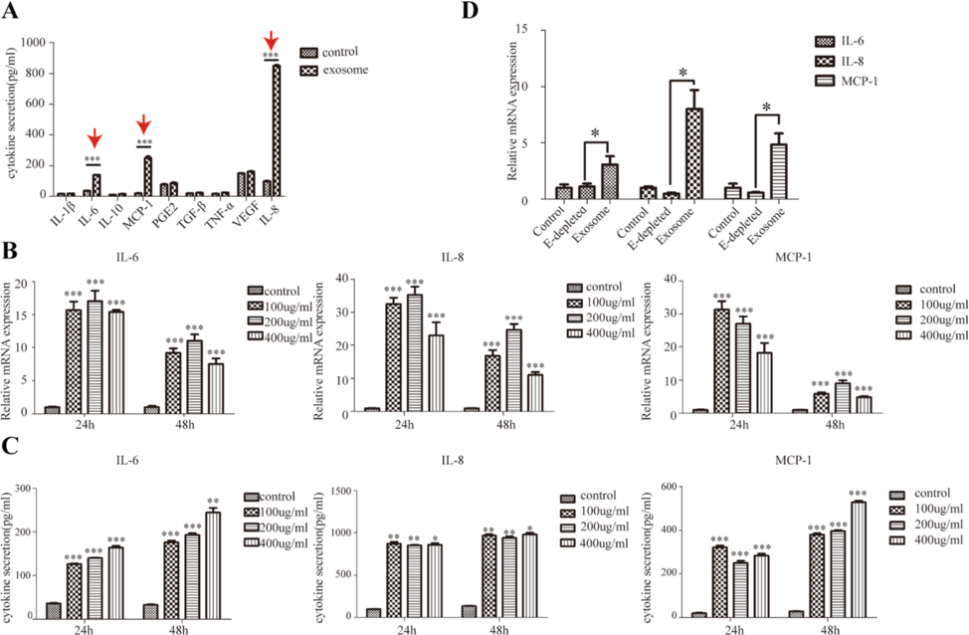
A549 cells derived exosomes significantly stimulate inflammatory cytokines production in MSCs. **a** Cytokine secretion profile of MSCs and exosomes-treated MSCs. MSCs were incubated with 200ug/ml exosomes for 24 h. **b**, **c** The mRNA level and protein level of IL-6, IL-8, and MCP-1 at different time points across a concentration gradient of exosomes (0, 100, 200, 400 μg/ml). **d** The mRNA expression changes of IL-6, IL-8 and MCP-1of MSCs treated with exosomes-depleted A549 cells culture medium (*P < 0.05, **P < 0.01, ***P < 0.001)

In our previous report about mRNA expression microarray [19], we noticed a remarkable elevation of genes associated with inflammation. Given the important link between inflammation and cancer progression, we began to explore whether A549 exosomes could change inflammatory response in MSCs. MSCs were exposed to 200 ug/ml A549 exosomes for 24 h, and the cytokine secretion profile of exosome-treated MSCs were detected by ELISA. Enhanced release of IL-6, IL-8, and MCP-1 was shown (Fig. 2a). To examine whether enhanced release of cytokines in exosome-treated MSCs was time- or concentration-dependent; we treated MSCs with different concentrations of A549 exosomes for 24 and 48 h. As shown in Fig. 2b for cytokine mRNA expression or Fig. 2c for cytokine protein release, no obvious time- or concentration-dependent manner was observed. To further confirm that the enhanced release of these cytokines was caused by exosome, we treated MSCs with exosome-depleted culture medium. As expected, enhanced expression of IL-6, IL-8, and MCP-1 was not observed (Fig. 2d). These data suggest that after exposure to cancer cell-derived exosomes, a new kind of pro-inflammatory MSCs (P-MSC) was generated.

### P-MSCs promote tumor growth in vivo

**Fig. 3:**
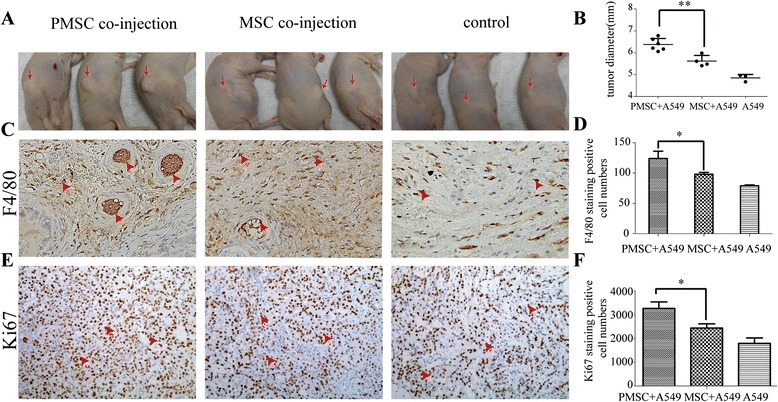
P-MSCs promote tumor growth in vivo. **a** Tumor promoting effects of P-MSCs in a xenograft mouse model. PMSC + A549, *n* = 6. 2 × 10^6^ A549 cells and 2 × 10^5^ exosomes-treated MSCs were injected. MSC + A549, *n* = 4. 2 × 10^6^ A549 cells and 2 × 10^5^ DF12-treated MSCs were injected. A549, *n* = 3, only 2 × 10^6^ A549 cells were injected. **b** the tumors in each group were dissected and tumor diameters were measured at 2 weeks after subcutaneous cell injection. **c** The macrophage infiltration was examined by F4/80 immunohistochemical staining of the tumor tissues harvested 2 weeks after A549 cell inoculation. Representatives of F4/80 staining from each group are shown (magnification 200×). **d** F4/80 + macrophages were quantitated and the data represent the mean number of F4/80+ macrophages per 200 × field (three fields per group). **e** Tumor cell proliferation was examined by Ki67 immunohistochemical staining of the tumor tissues. Representatives of Ki67 staining from each group are shown (magnification 200×). **f** Ki67 staining-positive cells were quantified and the data represent the mean number per 200 × field (three fields per group)

We next determined whether the altered cytokine expression in P-MSCs is accompanied by changes in tumor-promoting capacity in vivo. Using a nude mouse model, we subcutaneously injected A549 cells with P-MSCs, MSCs or PBS.As shown in Fig. 3a, the size of tumors significantly increased in the presence of P-MSCs in respect to tumor alone or tumor co-injected with unstimulated MSCs. Such stimulation in tumor growth was confirmed by measurement of tumor diameter (Fig. 3b). Since MCP-1-mediated macrophage recruitment was relevant with tumor growth and angiogenesis [20], the tumors were excised for examination of immune cell infiltration. We observed that F4/80 macrophages were much more abundant in tumors receiving P-MSCs than those receiving un-stimulated MSCs (Fig. 3c, d). This tumor-promoting effect and the macrophage-recruiting function of P-MSCs corresponded well to their high expression levels of MCP-1 (Fig. 2a), the major macrophage chemokines. In addition, Ki67 staining also showed elevated cell proliferation rate of A549 cells co-administered with PMSCs (Fig. 3e, f). Thus, MSCs educated by A549 exosomes gained an enhanced capacity in recruiting macrophages and in promoting tumor growth in vivo.

### NFκB signaling pathway is strikingly activated in P-MSCs

**Fig. 4:**
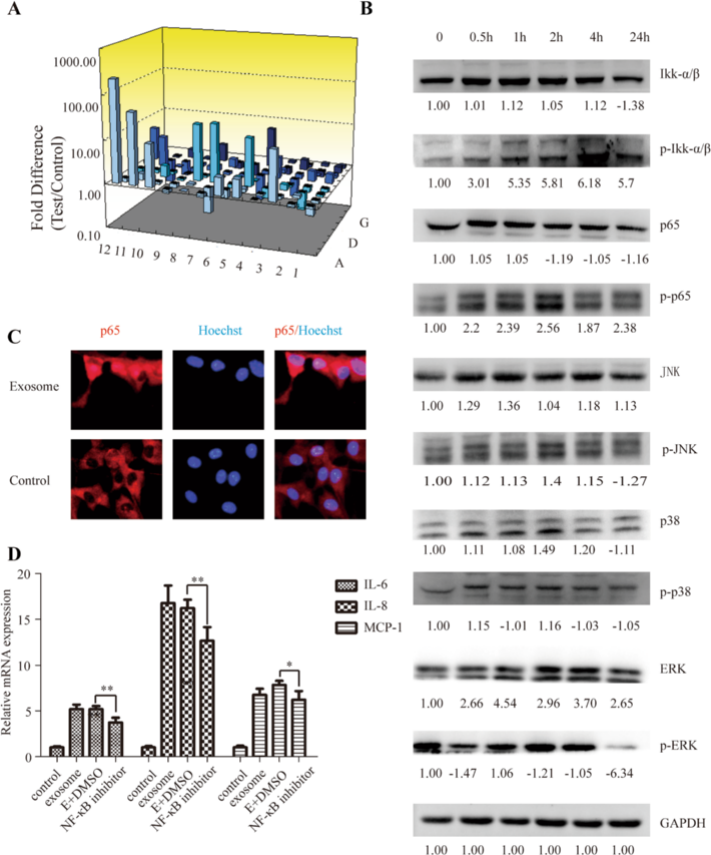
NFκB signaling pathway is strikingly activated in P-MSCs. **a** 3D profile of RT^2^ Profiler™ PCR Array of Human Toll-Like Receptor Signaling Pathway. **b** Activation of NF-κB in P-MSC was determined by Western blotting. The fold difference in band intensities was quantified and indicated under the image using the software of AlphaView. **c** Representative pictures of cellular immunofluorescence staining for nuclear p65 in MSCs or P-MSC at 2 h. Immunocytochemistry staining was performed using an anti-p65 antibody (*red*) and Hoechst (*blue*) for nuclear staining. **d** Relative mRNA expression of IL-6, IL-8, and MCP-1. MSCs were pre-treatment with or without the NF-κB inhibitor (PDTC, 10 μM) at 37 °C for 1 h and then incubated with 200 μg/ml A549 cell-derived exosomes at 37 °C for 24 h (*P < 0.05, **P < 0.01, ***P < 0.001)

To further investigate the signaling pathways involved in induction of PMSCs by A549 exosomes, we focused on TLR pathway as it has been reported to be closely related to inflammation. We analyzed gene-expression profiles of MSCs activated by or not by A549 exosomes through the use of a human toll-like receptor signaling pathway PCR array (see the Gene list at Table 1). According to the PCR array analysis (Fig. 4a), inflammation-related pathways including NFκB, JNK, and p38 signaling, were actually activated in P-MSCs. We then investigated the signaling events triggered by exosomes through Western blotting. As shown in Fig. 4b, a rapid activation of NFκB was detected by phosphorylation of Ikkα/β and the p65 subunit that peaked at 0.5–2 h after exosome exposure. Rapid activation of JNK, ERK, and p38 signaling was not detected (Fig. 4b). NFκB signaling activation involves phosphorylation of the p65 protein and translocation of the p65 protein to the cell nucleus. Therefore, we determined the localization of p65 in MSCs before and after exposure to A549 exosomes. As expected, A549 exosome stimulation resulted in a significant translocation of p65 from a cytoplasmic to a nuclear localization in Fig. 4c. Moreover, we demonstrated that NFκB inhibitor PDTC remarkably suppressed the enhanced expression of IL-6, IL-8, and MCP-1 induced by A549 exosomes in MSCs (Fig. 4d). Together, these results suggest that lung tumor-derived exosomes induce the generation of P-MSCs through activation of NFκB signaling pathway.

**Table 1:** Gene lists of human toll-like receptor signaling pathway and data for 3D profile

*3D profile gene data*	*A*	*B*	*C*	*D*	*E*	*F*	*G*	*H*
**1**	BTK	ECSIT	IKBKB	IRF3	MYD88	RELA	TLR3	ACTB
	0.64	0.86	0.88	0.84	1.63	1.17	1.39	1.14
**2**	CASP8	EIF2AK2	IL10	JUN	NFKB1	RIPK2	TLR4	B2M
	0.86	1.16	0.41	0.75	1.77	1.06	0.74	1.16
**3**	CCL2	ELK1	IL12A	LTA	NFKB2	SARM1	TLR5	GAPDH
	13.80	0.85	1.35	0.91	0.41	0.81	1.71	0.87
**4**	CD14	FADD	IL1A	LY86	NFKBIA	SIGIRR	TLR6	HPRT1
	1.00	0.91	0.60	0.82	3.01	1.19	0.68	1.09
**5**	CD180	FOS	IL1B	LY96	NFKBIL1	TAB1	TLR7	RPLPO
	2.57	1.18	12.43	1.02	0.27	0.83	7.73	0.79
**6**	CD80	HMGB1	IL2	MAP2K3	NFRKB	TBK1	TLR8	
	2.57	0.65	0.82	1.10	0.86	0.71	0.55	
**7**	CD86	HRAS	IL6	MAP2K4	NR2C2	TICAM1	TLR9	
	0.82	0.27	21.55	0.95	0.85	1.58	0.71	
**8**	CHUK	HSP1A1	IL8	MAP3K1	PELT1	TICAM2	TNF	
	1.01	1.12	19.37	0.89	1.23	0.75	2.50	
**9**	CLEC4E	HSPD1	IRAK1	MAP3K7	PPARA	TIRAP	TNFRSF1A	
	0.82	0.82	0.91	0.96	0.79	0.88	1.07	
**10**	CSF2	IFNA1	IRAK2	MAP4K4	PRKRA	TLR1	TOLLIP	
	9.57	0.89	1.79	0.87	1.03	0.48	1.58	
**11**	CSF3	IFNB1	IRAK4	MAPK8	PTGS2	TLR10	TRAF6	
	43.95	1.42	1.58	0.86	4.75	0.30	0.91	
**12**	CXCL10	IFNG	IRF1	MAPK8IP3	REL	TLR2	UBE2N	
	217.01	0.82	2.03	0.74	1.19	5.64	0.94	

MSCs were treated with or without 200 μg/ml A549 cell-derived exosomes for 24 h. Total RNA were analyzed using human toll-like receptor signaling pathway array. Fold change of RNA expression was used for 3D profile (200–24 h/0–24 h)

### Exosomes trigger cytokine production in MSCs through TLR2

**Fig. 5:**
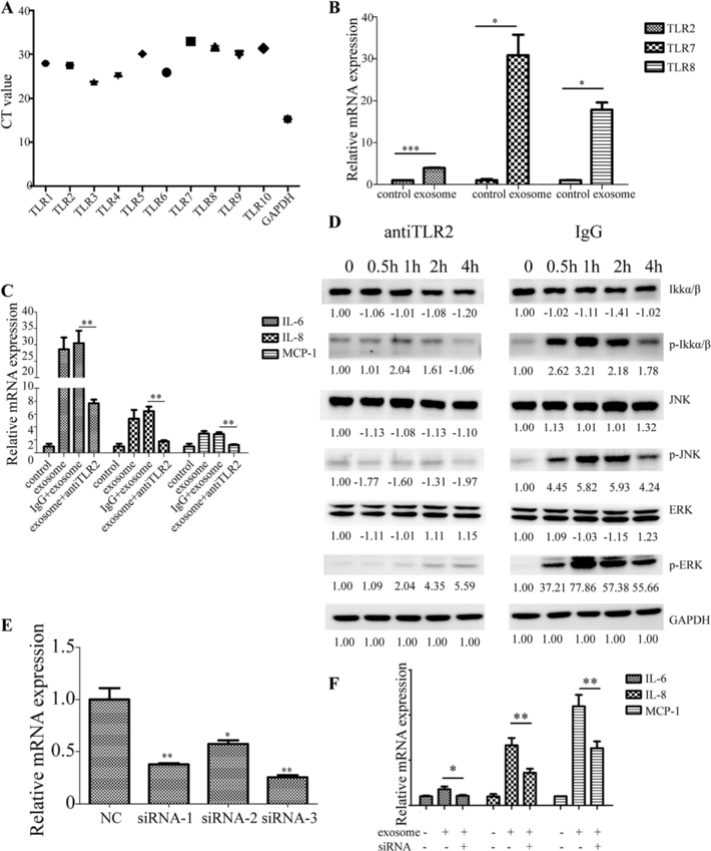
Exosomes trigger cytokine production in MSCs through TLR2. **a** The expression level of TLR 1 ~ 10 of MSCs. GAPDH was used as control. **b** mRNA expression changes of TLR2, 7, 8 were detected by RT-PCR after treatment with exosomes. **c** mRNA expression changes of IL-6, IL-8, and MCP-1 of MSCs. MSCs were pre-treated withTLR2 neutralizing antibody or mouse IgG2b isotype control at 37 °C for 1 h and then incubated with exosomes for 24 h. **d** Western blotting analysis of NF-κB activation in P-MSCs. MSCs treated with mouse IgG2b isotype control was used as control. **e** The interfering efficiency of three pairs of TLR2 siRNAs was measured by RT-PCR. **f** mRNA expression changes of IL-6, IL-8, and MCP-1. MSCs were stimulated with exosomes after knockdown of TLR2 by siRNA for 24 h (*P < 0.05, **P < 0.01, ***P < 0.001)

The effects of exosomes on NFκB prompted us to study more closely the role of TLRs in exosome-mediated signaling. RT-PCR results showed that all TLRs were detectable within 33 cycles in MSCs (Fig. 5a). To determine which TLR was specifically responsible for tumor exosome-mediated cytokine up-regulation in MSCs, we first detected TLRs changes after exosome stimulation. We found that TLR2, TLR7, and TLR8 mRNA expression was significantly increased after exosomes stimulation (Fig. 5b). Here, we focused on TLR2 for three reasons. Firstly, MSCs have higher expression of TLR2 than TLR7 or TLR8 as demonstrated in Fig. 5a, although up-regulation of TLR7 and TLR8 is more significant (Fig. 5b). Secondly, TLR2 is localized on cell plasma membrane whereas TLR7 or TLR8 resides within endosomal compartments. Lastly, TLR2 can recognize lipopeptides while both TLR7 and TLR8 were shown to sense single-stranded viral RNA [21–23]. Using a TLR2 neutralizing antibody, we found that blockade of TLR2 in MSCs decreased the expression of inflammatory factors induced by lung tumor-derived exosomes and inhibited activation of NFκB signaling pathway (Fig. 5c, d).

Furthermore, we knocked down TLR2 with three pairs of designed siRNAs, and we chose siRNA3 as it had the highest interference efficiency of nearly 75 % (Fig. 5e).

SiRNA knockdown of TLR2 also attenuated the expression of IL-6, IL-8 and MCP-1 (Fig. 5f, Additional file 1). Collectively, these data implicated that TLR2 was relevant for exosomes-mediated increase of cytokines IL-6, IL-8, and MCP-1 in MSCs.

### Hsp70 on exosomes contribute to cytokine production through TLR2

**Fig. 6:**
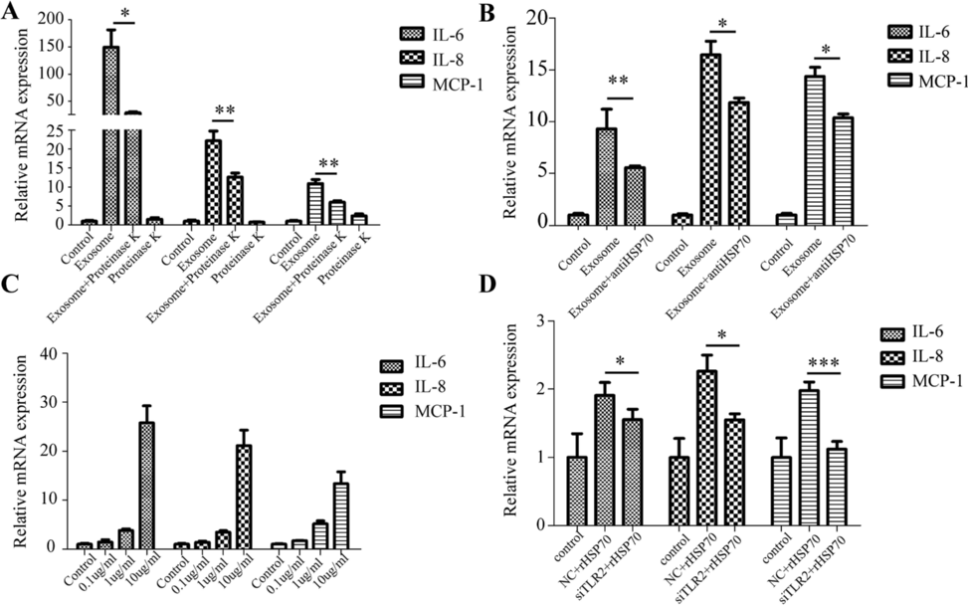
Hsp70 on exosomes contribute to cytokine production through TLR2. **a** mRNA expression changes of IL-6, IL-8, and MCP-1. Exosomes were pre-treated with proteinase K (5 μg/ml) at 37 °C for 1 h and then treated at 95 °C for 10 min after proteinase K treatment to abolish its activity. Proteinase K treatment alone was used as another control. **b** mRNA expression changes of IL-6, IL-8, and MCP-1. Exosomes were pre-treated with or without anti-HSP70 antibody at 37 °C for 1 h. 10 μg/ml anti-HSP70 was used for blocking. **c** mRNA expression changes of IL-6,IL-8, and MCP-1 in MSCs treated with different concentrations of rHSP70 proteins for 24 h. **d** mRNA expression changes of IL-6, IL-8, and MCP-1 in MSCs treated with rhHSP70 after knockdown of TLR2 by siRNA for 24 h (*P < 0.05, **P < 0.01, ***P < 0.001)

To find out whether proteins on exosomes were responsible for PMSCs induction, we treated exosomes with proteinase K before exposure to MSCs. Interestingly, diminished expression of IL-6, IL-8, andMCP-1 could be observed after treatments with proteinase K (Fig. 6a), suggesting that proteins on exosomes were related with MSCs cytokine production. A variety of molecules have been classified as TLR2 ligands including Versican, HMGB1, and heat shock proteins (HSP). HSP70 is highly expressed on A549 exosomes and it was reported to be a TLR2 ligand [24]. We then evaluated whether A549 exosomes induced generation of P-MSCs through HSP70 or not. Neutralizing antibody of HSP70 could partially suppress the enhanced expression of IL-6, IL-8, and MCP-1 induced by A549 exosomes in MSCs (Fig. 6b). Recombinant human HSP70 (rHSP70) added to MSCs culture mimicked the effect of A549 exosomes in a dose-dependent manner (Fig. 6c). Moreover, interfering TLR2 expression with siRNA could attenuate the effects caused by rHSP70 (Fig. 6d). Altogether, these results indicate that Hsp70 expressed on the surface of exosomes could trigger TLR2 signaling in MSCs.

## Discussion

Exosomes, first considered as “garbage” released from cells, are now exerting starring roles in intercellular communication. Research about exosomes focuses on three areas: disease biomarker in early stage, membrane vesicles as conveyors of immune responses, and roles in cancer [25]. Here, we focus on exosomes in tumor microenvironment. MSCs can be recruited into tumors and form a major component of the tumor microenvironment. Emerging evidence show that tumor cells could modulate MSCs through exosomes including prostate cancer, melanoma, cancer and so on [26, 27]. Although many cancer cells could modulate MSCs into myofibroblasts, the molecular mechanisms involved are not the same. One group reported that the tumorigenic reprogramming of MSCs in prostate cancer was associated with exosomal oncogenic factors such as H-ras and K-ras transcripts, oncomiRNAs, miR-125b etc. [28] Another group suggested that prostate cancer cells could trigger differentiation of fibroblasts into myofibroblasts through exosomal TGF-β [26]. Breast cancer-derived exosomes induced the myofibroblastic phenotype and functionality in AMSCs via the SMAD-mediated signaling pathway [10].

Here, we found that MSCs could be educated by lung cancer cell-derived exosomes into pro-inflammatory phenotype and therefore got tumor supportive characteristics. Different from the current research tendency which focuses on the transition from MSCs to myofibroblast caused by tumor cell-derived exosomes, we emphasize on the inflammation phenotype of MSCs. Our results were consistent with previous clinical findings that MSCs require pro-inflammatory cytokines to induce their immunosuppressive function [29, 30]. This indicates the importance of pro-inflammatory MSCs in tumors progression.

However, few studies have elucidated mechanisms about which signal pathways trigger pro-inflammatory phenotype changes in MSCs. Jerome Paggetti reported that exosomes released by chronic lymphocytic leukemia could induce an inflammatory phenotype in stromal cells through activating AKT, ERK1/2, CREB, and GSK3α/β signaling pathways [12]. Several related research explained how tumor cell-derived exosomes cause inflammation in immune cells. Sanchita Bhatnagar et.al found that exosomes released from bacteria-infected macrophages stimulated a pro-inflammatory response in a toll-like receptor—and myeloid differentiation factor 88 (MyD88)—dependent manner [31]. Fanny Chalmin demonstrated that mice tumor-derived exosomal HSP72 induce IL-6 production by MDSCs through activation of TLR2 and its adaptor MyD88, leading to Stat3 phosphorylation and the promotion of MDSC immunosuppressive functions [32]. Some others thought stress-induced Hsp72 activates macrophages, dendritic cells, and neutrophils by binding to surface bound TLR4 and induce the secretion of pro-inflammatory cytokines [33]. Alexzander Asea et al. [34] also illustrated that HSP70 utilized both TLR2 and TLR4 to transduce its pro-inflammatory signal in a CD14-dependent fashion.

These studies suggested the involvement of HSP proteins in exosome-associated inflammation. There remains a question—whether pro-inflammatory properties are unique to cancer-derived exosomes or are an intrinsic feature of all exosomes. We hypothesized that exosomes have a common determinant, HSP70, which is one of the inducers of P-MSCs. However, other protein components of exosomes may also be involved in this process [35]. These determinants need to be further characterized.

In our study, we found that HSP70 on the surface of A549 cell-derived exosomes could activate NF-κB signaling through TLR2 on MSCs. However, TLR2 neutralizing antibody could partially reduce the effects of exosomes, suggesting that TLR4, which also can interact with HSP proteins, may serve as a substitute for TLR2.

Additionally, we should consider the role of RNA component in exosomes in our future study. We noticed the decreased expression of inflammatory factors after pre-treating exosomes with Rnase A (data not shown), which implied the involvement of exosomal RNAs. It has been shown that acute stressor exposure modified plasma exosome-associated Hsp72 and microRNA (miR-142-5p and miR-203) [36]. Exosomal miRNAs can affect cells of the tumor microenvironment [37] both in a canonical (mRNA-targeting) and non-canonical (receptor-binding) manner. Tumor exosomal miR-21 and miR-29a can function by binding as ligands to receptors of the TLR family in immune cells, triggering a TLR-mediated prometastatic inflammatory response that ultimately leads to tumor growth and metastasis [38].

Considerable evidence suggests that pro-inflammatory pathways drive self-renewal of cancer stem-like cells. HSPs are key players during inflammation besides their chaperone and cytoprotective functions. Interest in HSP70 inhibitors is increasing as potential anticancer agents in recent years [39, 40]. By reprogramming pro-inflammatory MSCs in tumor microenvironment using HSP70 inhibitors, tumor progression may be controlled. But there is still a lot of “dark matter” to reveal before the welcome of light for cancer patients.

## Conclusions

**Fig. 7:**
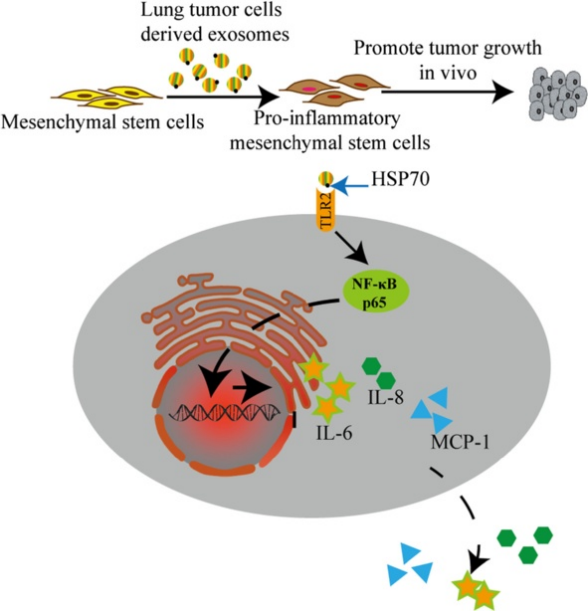
A schematic illustration showed that lung tumor cell-derived exosomes could educate naïve MSCs into a new kind of pro-inflammatory MSCs (P-MSCs) by activating TLR2/NF-κB signaling through exosomal surface HSP70

This study demonstrates that lung cancer cell-derived exosomes could educate naïve MSCs into a new kind of pro-inflammatory MSCs (P-MSCs) by activating TLR2/NF-κB signaling through exosomal surface HSP70. Importantly, this new kind of P-MSCs could support tumor growth (Fig. 7).

## Methods

### Exosome isolation

Exosome extraction was performed as previously described [41]. Briefly, A549 cells were cultured in serum-free DF12 medium for 24 h. Then, the culture medium was collected and centrifuged at 800*g* for 5 min and additional 2000*g* for 10 min to remove lifted cells. The supernatant was subjected to filtration on a 0.1-mm-pore polyethersulfone membrane filter (Corning) to remove cell debris and large vesicles, followed by concentration by a 100,000 Mw cutoff membrane (CentriPlus-70, Millipore). The volume of supernatant was reduced from approximately 250–500 mL to less than 5 mL. The supernatant was then ultracentrifuged at 100,000*g* for 1 h at 4 °C using 70Ti rotor (Beckman Coulter). The resulting pellets were resuspended in 6 mL PBS and ultracentrifuged at 100,000 *g* for 1 h at 4 °C using 100Ti rotor (Beckman Coulter).

### Transmission electron microscopy

Purified exosomes were fixed with 1 % glutaraldehyde in PBS (pH 7.4). After rinsing, a 20-uL drop of the suspension was loaded onto a formvar/carbon-coated grid, negatively stained with 3 % (w/v) aqueous phosphotungstic acid for 1 min, and observed by transmission electron microscope.

### Isolation and culture of MSCs from adipose tissue

Human adipose tissue was obtained from liposuction aspirates with informed consent of the donors and was performed according to procedures provided by the Ethics Committee at the Chinese Academy of Medical Sciences and Peking Union Medical College. The isolation and culture procedures were described as previously reported [42]. hAMSCs were resuspended in 12 ml culture medium and seeded at a density of 2 × 10^6^ cells in a 75-cm^2^ culture flask. Cell cultures were maintained at 37 °C in a humidified incubator with 5 % CO_2_ and passaged with trypsin/EDTA when cells were confluent. Passage 3 cells were used for following experiments.

### Quantitative real-time polymerase chain reaction

Cultured cells were lysed by TRIzol (Invitrogen, USA), and RNA was extracted according to the manufacturer’s instruction. One microgram of total RNA from each sample was reverse transcribed using M-MLV (Takara) in a final volume of 20 uL. The polymerase chain reaction (PCR) amplification was carried out using the Step-one System (Bio-Rad) with SYBR Green Mastermix (Takara). All quantitative real-time PCR (qRT-PCR) results were carried out in duplicate and normalized to GAPDH. The primer of the related gene list is found in Table 2.

**Table 2:** Primers for RT-PCR

Gene	Forward primer and reverse primer (5'–3')
*ALP*	CCACGTCTTCACATTTGGTG; AGACTGCGCCTGGTAGTTGT
*RUNX2*	TGTCATGGCGGGTAACGAT; AAGACGGTTATGGTCAAGGTGAA
*OCN*	GGCGCTACCTGTATCAATGG; GTGGTCAGCCAACTCGTCA
*PPAR γ*	CCTATTGACCCAGAAAGCGATT; CATTACGGAGAGATCCACGGA
*LPL*	ACAAGAGAGAACCAGACTCCAA; AGGGTAGTTAAACTCCTCCTCC
*C/EBPβ*	CTTCAGCCCGTACCTGGAG; GGAGAGGAAGTCGTGGTGC
*IL-6*	ACTCACCTCTTCAGAACGAATTG;CCATCTTTGGAAGGTTCAGGTTG
*IL-8*	ACTCCAAACCTTTCCACCCC; TTCTCAGCCCTCTTCAAAAACTTC
*MCP-1*	CAGCCAGATGCAATCAATGCC; TGGAATCCTGAACCCACTTCT
*IL-1β*	AGCTACGAATCTCCGACCAC; CGTTATCCCATGTGTCGAAGAA
*TNF-α*	CCTCTCTCTAATCAGCCCTCTG; GAGGACCTGGGAGTAGATGAG
*IFN-α*	GCCTCGCCCTTTGCTTTACT; CTGTGGGTCTCAGGGAGATCA
*IFN-β*	GCTTGGATTCCTACAAAGAAGCA;ATAGATGGTCAATGCGGCGTC
*IFN-γ*	TCGGTAACTGACTTGAATGTCCA; TCGCTTCCCTGTTTTAGCTGC
*ACTA2*	CGATGCTCCCAGGGCTGTTT; TTCGTCACCCACGTAGCTGTCTTT
*TLR1*	CCACGTTCCTAAAGACCTATCCC; CCAAGTGCTTGAGGTTCACAG
*TLR2*	ATCCTCCAATCAGGCTTCTCT; GGACAGGTCAAGGCTTTTTACA
*TLR3*	TTGCCTTGTATCTACTTTTGGG; TCAACACTGTTATGTTTGTGGGT
*TLR4*	AGACCTGTCCCTGAACCCTAT; CGATGGACTTCTAAACCAGCCA
*TLR5*	TCCCTGAACTCACGAGTCTTT; GGTTGTCAAGTCCGTAAAATGC
*TLR6*	TGAATGCAAAAACCCTTCACC; CCAAGTCGTTTCTATGTGGTTGA
*TLR7*	CACATACCAGACATCTCCCC; CCCAGTGGAATAGGTACACAGTT
*TLR8*	ATGTTCCTTCAGTCGTCAATGC; TTGCTGCACTCTGCAATAACT
*TLR9*	CTGCCACATGACCATCGAG; GGACAGGGATATGAGGGATTTGG
*TLR10*	GGTTCTTTTGCGTGATGGAATC; GTCGTCCCAGAGTAAATCAAC
*GAPDH*	GGTCACCAGGGCTGCTTTTA; GGATCTCGCTCCTGGAAGATG

### Western blotting

After washing twice with cold PBS, cells were lysed in RIPA lysis buffer (Beyotime, Shanghai, China) with 1 mM PMSF and protease inhibitor cocktail on ice for 30 min, manually scraped from culture plates and then quantified using the BCA Protein Assay Kit (Beyotime). Proteins were separated on 10 % sodium dodecyl sulfate–polyacrylamide gel electrophoresis (SDS-PAGE) gels, electroblotted onto a polyvinylidene difluoride (PVDF) membrane (0.22 μm, Millipore, Billerica, MA, USA). The membranes were blocked with 5 % BSA and incubated with specific antibodies overnight at 4 °C and then were incubated with horseradish peroxidase (HRP)-conjugated secondary antibody for 1 h at room temperature. The primary antibodies were as follows: IKKα/β, phosphorus IKKα/β, p65, phosphorus p65, JNK, phosphorus JNK, phosphorus p38 (1/1000, Cell Signaling Technology, USA), CD63, HSP70 (1/1000, Abcam), GAPDH (1/1000, Santa cruz), and β-actin (1/1000, Zhongshan, Beijing China). Secondary (HRP)-conjugated antibodies were purchased from NeoBioscience. Antibody and antigen complexes were detected using chemiluminescent ECL reagent (Millipore, USA).

### siRNA transfection

Three pairs of siRNA of TLR2 were designed and synthesized (Gene Pharma, Inc., Shanghai, China). The synthetics were transfected into AMSCs at the final concentration of 200 nM using lipofectamine 2000 (Invitrogen, USA) according to the manufacturer’s instructions. The whole transfection process was proceeded in a non-serum medium named opti-mem (Gibco, USA) for 6 h at 37 °C in a humidified environment containing 5 % CO2. After transfection, the medium was changed into DF12 medium with or without cancer exosomes.

### Cytokine analysis

Culture supernatants were collected after treatment with or without exosomes for 24 h. The concentrations of all cell cytokines in supernatants were measured using ELISA kits (BD Technologies).

### Immunofluorescence staining

The cultured cells were fixed at 4 °C in ice-cold methanol for 10 min, washed three times in phosphate-buffered saline (PBS), and then permeabilized in 0.1 % Triton X-100/PBS for 10 min at room temperature. Nonspecific binding was blocked with 0.5 % Tween-20/PBS containing 1 % bovine serum albumin (BSA) for 30 min. The primary antibodies were incubated at 4 °C overnight. The secondary antibodies incubated for 1 h at room temperature. The incubated cells were washed in PBS, and Hoechst 33342 (Sigma-Aldrich) was used to visualize nuclei. p65 antibody (10745-1-AP) was purchased from Proteintech.

### Animal experiments

All nude mice were purchased from the Laboratory Animal Center of the Chinese Academy of Medical Sciences (Beijing, China). All mice were bred and maintained under specific pathogen-free conditions. Animal use and experimental procedures were approved by the Animal Care and Use Committee of the Chinese Academy of Medical Sciences. All nude mice received a subcutaneous injection of 2 × 10^6^ A549 cells. One group received a subcutaneous injection of 2 × 10^5^ PMSCs. The other group received a subcutaneous injection of 2 × 10^5^ MSCs. The last group only received an injection of A549 cells. The tumor volume was measured after 2 weeks. The tumor tissues were fixed with 10 % PFA and the peripheral blood were collected for C-flow analysis. Each group was treated with HE, Ki67, and F4/80 staining. Ki67 antibody was purchased from Proteintech (19972-1-AP). F4/80 antibody was purchased from Abcam (ab6640).

### Statistical analysis

Data are presented as mean ± SD. Comparisons between groups were analyzed via Student’s *t* test. Differences were considered statistically significant at *P < 0.05, **P < 0.01, and ***P < 0.001.

## Additional file

Additional file 1: mRNA expression changes of IL-6, IL-8 and MCP-1 in MSCs stimulated with exosomes after knockdown of TLR2 by siRNA combinations for 24h. (TIF 236 kb)

## Abbreviations

ALP: alkaline phosphatase
AMSCs: adipose tissue-derived MSCs
BMSCs: bone-marrow-derived MSCs
EDTA: ethylene diamine tetra-acetic acid
ELISA: enzyme-linked immunosorbent assay
FBS: fetal bovine serum
FITC: fluorescein isothiocyanate
H-DMEM: high glucose of Dulbecco’s modified Eagle’s medium
HE: hematoxylin-eosin
HSP: heat shock protein
IL: interleukin
JNK: c-Jun N-terminal kinase
MCP-1: monocyte chemotactic protein 1
MSCs: mesenchymal stem cells
MyD88: myeloid differentiation factor 88
NF-κB: nuclear factor kappa-light-chain-enhancer of activated B cells
PAMP: pathogen associated molecular pattern
PBS: phosphate buffered saline
PCR: polymerase chain reaction
P-MSC: pro-inflammatory MSCs
qRT-PCR: quantitative real-time PCR
RT-PCR: reverse transcription polymerase chain reaction
TGF-β1: transforming growth factor beta 1
TLR: toll-like receptor
