# Supplementary figures and images for: Lung tumor exosomes induce a pro-inflammatory phenotype in mesenchymal stem cells via NFκB-TLR signaling pathway
Source: J Hematol Oncol. 2016 Apr 18;9:42. doi: 10.1186/s13045-016-0269-y (PMC4836087; doi:10.1186/s13045-016-0269-y)

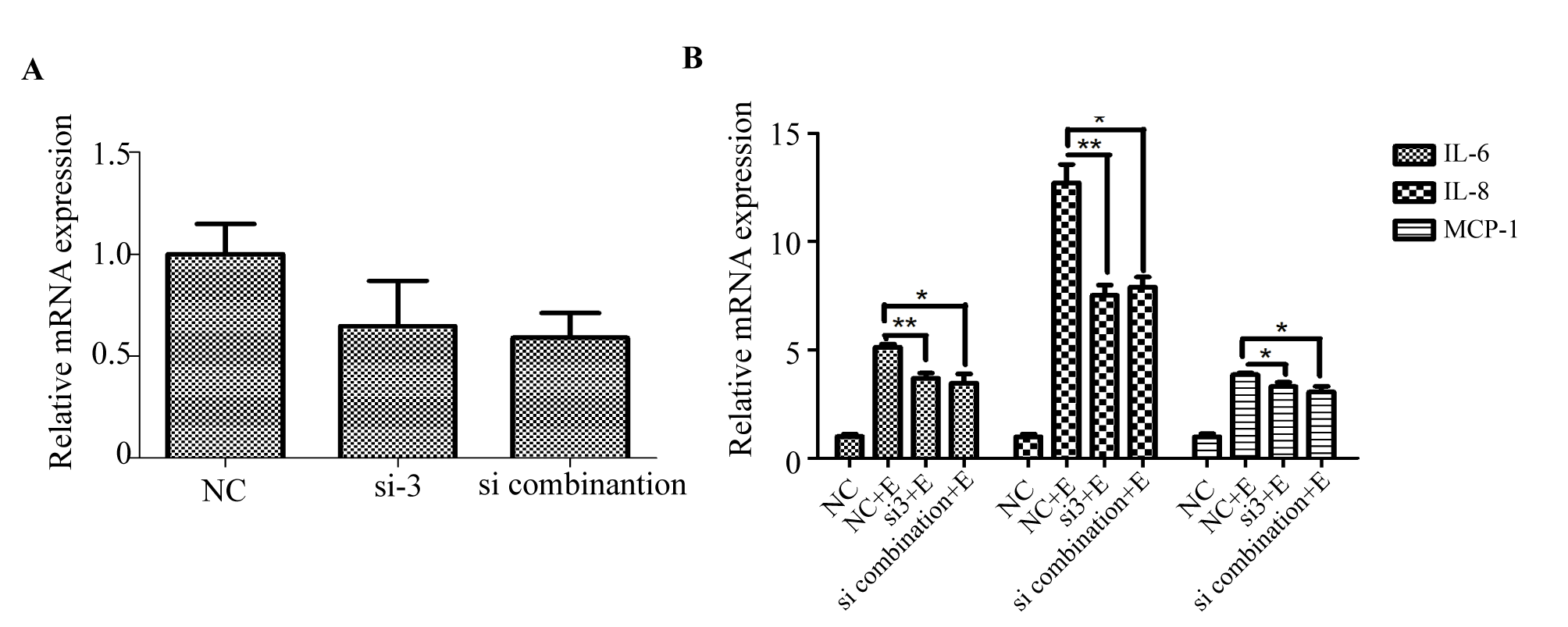

Supplement: Additional file 1: — mRNA expression changes of IL-6, IL-8 and MCP-1 in MSCs stimulated with exosomes after knockdown of TLR2 by siRNA combinations for 24h. (TIF 236 kb) [file 13045_2016_269_MOESM1_ESM.tif]
